# Probiotic Fermented Feed Alleviates Liver Fat Deposition in Shaoxing Ducks *via* Modulating Gut Microbiota

**DOI:** 10.3389/fmicb.2022.928670

**Published:** 2022-07-13

**Authors:** Tiantian Gu, Mingcai Duan, Ruikun Zhang, Tao Zeng, Wenwu Xu, Weifeng Feng, Chunqing Jiang, Yong Tian, Li Chen, Lizhi Lu

**Affiliations:** ^1^State Key Laboratory for Managing Biotic and Chemical Threats to the Quality and Safety of Agro-Products, Institute of Animal Science and Veterinary, Zhejiang Academy of Agricultural Science, Hangzhou, China; ^2^Institute of Animal Husbandry and Veterinary Medicine, Zhejiang Academy of Agricultural Sciences, Hangzhou, China; ^3^Jinhua Jinwu Agricultural Development Co., Ltd, Jinhua, China

**Keywords:** probiotic fermented feed, liver fat deposition, transcriptomics, 16s rDNA, gut microbiota, ducks

## Abstract

The aim of this study was to investigate the effects of different probiotic fermented feed (PFF) on ameliorating liver fat accumulation by modulating the gut microbiota. A total of 216, 120-day-old Shaoxing ducks were divided into three groups, including the control group (basal diet), or the basal diet supplemented with 25 or 35% PFF. The results of the animal experiment showed that supplementation with PFF markedly alleviated the formation of liver and abdominal lipid droplet and decreased the levels of serum triglyceride (TG) in Shaoxing ducks. 16s rDNA showed that PFF could modulate the composition of gut microbiota, in particular, modulating the ratio of Firmicutes to Bacteroidetes. Moreover, PFF restructures the gut microbiome by reducing the abundance of Ruminococcaceae, Lachnospiraceae, and Prevotellaceae in ducks. Additionally, liver transcriptome analysis indicated that the PFF supplementation significantly downregulated the mRNA expression of peroxisome proliferator-activated receptor gamma *(PPARG)*, acyl-CoA desaturase *(SCD), DBI*, fatty acid synthase *(FASN)*, ELOVL fatty acid elongase 2 (*ELOVL2), ELOVL6*, and hydroxysteroid 17-beta dehydrogenase (*HSD17B12)* and upregulated the mRNA expression of *CPT1B*, which was widely associated with lipid metabolism processes, such as fatty acid elongation, PPAR signaling pathway, and ether lipid metabolism. Correlation analysis indicates that the expression changes of liver metabolism-related genes by PFF are highly correlated with the Ruminococcaceae, Lachnospiraceae, and Prevotellaceae levels. These findings demonstrated that PFF supplementation modulates gut microbial composition to activate liver lipid metabolism-related genes, which results in less lipid deposition in ducks. These findings provide novel insights into the molecular mechanisms of dietary PFF underlying liver fat accumulation by regulating gut microbiota.

## Introduction

Duck liver has a unique ability of fat accumulation, while excessive liver fat deposition may easily lead to liver injury, which will cause a reduction in production (Zhang et al., 2020b). According to new evidence, nutritional supplement plays a dominant role in managing and balancing lipid metabolism (Anhê et al., 2019), which allows ducks to preferentially protect themselves from excessive liver lipid deposition when they are fed with nutrition diets to improve production performance in duck industries. Therefore, novel treatments with dietary are necessary to control liver fat accumulation.

Fermented products, which provided live microbial, have been commonly applied in the human and poultry industry (Li et al., 2020; Park et al., 2021; Wu et al., 2021; Lv et al., 2022). Rice bran fermented by *Weissella koreensis* DB1 can decrease the serum levels of triglyceride (TG) and total cholesterol (TC) and significantly downregulate the hepatic gene expression of C/EBP, SREBP-1c, FAS, and ACC in mice (Park et al., 2021). Meanwhile, many *in vitro* fermentation studies have shown that fermented feed can improve poultry egg performance, immune function, intestinal development, and lipid metabolism (Engberg et al., 2009; Gao et al., 2009; Olukomaiya et al., 2019; Sugiharto and Ranjitkar, 2019; Saleh et al., 2021). Laying hens fed with fermented feed can improve egg weight, shell weight, and shell stiffness (Engberg et al., 2009). Fermented diets can enhance the intestinal digestive function and morphology to modulate the gut microbial composition in broilers (Gao et al., 2009). In addition, broilers fed with fermented feed significantly increased the feed-to-gain ratio and reduced the hepatic gene expression of PPARγ (Saleh et al., 2021). Thus, feeding fermented feeds is beneficial to the health of poultry. However, at present, less information has been published on the function of probiotic fermented feed (PFF) in the duck liver fat deposition. Increasing evidence has suggested that gut microbiota plays an essential role in controlling liver lipid metabolism (Ley et al., 2006; Clemente et al., 2012; Ma et al., 2019), and it is receiving great attention that fermented diets have wide therapeutic effects in improving lipid metabolism through regulating intestinal environment (Aron-Wisnewsky et al., 2021). Kimchi has found it to alter gut microbiota composition and attenuate metabolic syndrome in Korean women with obesity (Han et al., 2015). The mice with non-alcoholic fatty liver disease (NAFLD) feeding with fermented pickles altered the gut environment and decreased liver fat absorption (Mu et al., 2020). Thus, the fermented diet has been put forward as a key player in alleviating fat deposition by ameliorating the gut microbial composition. However, whether PFF can ameliorate duck liver fat deposition by modulating the gut microbiota remains unclear.

In this study, we systematically evaluated the effects of the supplementation with PFF applied with RNA-seq transcriptome analysis and 16s rDNA sequencing to define the mechanisms involving liver lipid accumulation effects in Shaoxing ducks.

## Methods

### Ethical Statement

All samplings were approved by the Zhejiang Academy of Agricultural Sciences' Animal Care Committee.

### Animals and Experimental Design

The experimental animals were female Shaoxing ducks (*Anas platyrhynchos*) obtained from Jinhua Jinwu Agricultural Development Co., Ltd., Jinhua, China, and a total of 216, 120-day-old Shaoxing ducks were randomly divided into three groups with 6 replicates containing 12 ducks for each group. The three treatment groups were allocated to either the control group (basal diet, control) or the basal diet supplemented with 25 or 35% PFF (25% PFF and 35% PFF, respectively). The experimental period was 90 days. The fermented feed was obtained using the *Lactobacillus, Yeast*, and *Bacillus* complex on the basis of basal diet under liquid fermentation. The commercial basal diet of Shaoxing ducks was purchased from Shanghai Nonghao Feed Co., Ltd., and the composition and nutritional level of the basic diet is shown in Supplementary Table 1. Ducks were raised alone per cage (28 × 40 × 40 cm) with nipple drinkers and tubular feeders. They were given natural light and artificial light for a period of 17 h a day. The room temperature was kept at flowing air for adaptation. All experimental ducks were healthy and were not administered any antibiotic treatments during the experiment.

### Sample Collection

At 210 days of age, blood was obtained from the wing veins of each ducks and serum was collected after centrifugation at 3,000 r/min for 10 min at 4°C and then stored at −80°C until use. After blood collection, ducks were immediately euthanized with sodium pentobarbital [150 mg/kg body weight (BW)] and killed by exsanguination. The liver and abdominal fat was fixed with 4% buffered formaldehyde for Oil Red O Staining analysis. The liver and cecal chyme were collected and snap-frozen in liquid nitrogen and stored at −80°C for RNA-seq and 16s analysis, respectively.

### Serum TC, TG, LDL-C, and HDL-C Determination

The concentrations of serum TC, TG, high-density lipoprotein cholesterol (HDL-C), and low-density lipoprotein cholesterol (LDL-C) were measured using commercial kits (Nanjing Jiancheng Bioengineering Institute, China) according to the instructions in the kits.

### Oil Red O Staining

The liver and abdominal fat tissues were sectioned at 10 μm for Oil-Red O staining (room temperature) to assess lipid droplet formation. The sections were stained with Oil Red O solution for 10 min. Then, distilled water was used to wash off the solution. Hematoxylin was restained to incubate with the sections for an additional 2 min and then washed with distilled water (10 min), dried, and embedded with the aqueous medium. The slices were observed using a Nikon E100 microscope, and pictures were taken at a magnification of 400×.

### Evaluation of the Liver Transcriptome

Total RNA was extracted using RNAiso Plus regent (TAKARA, Japan) following the protocol of the manufacturer. RNA integrity and concentration was carried out with the 2100 Bioanalyzer (Agilent, USA). The liver cDNA library was conducted on the Illumina sequencing platform using Illumina HiSeq 2000 (Illumina, USA) and 150-bp paired-end reads were produced. Raw data were refined and remove index, adapter, and low-quality sequences were used to obtain clean data with high-quality reads. The obtained data were compared with the *A. platyrhynchos* genome reference PK_2015 (https://www.ncbi.nlm.nih.gov/genome/2793genome_assembly_id=426073). Data with absolute fold change (FC) > 2.0 and *p* < 0.05 (*t*-test) were considered differentially expressed transcripts. The differentially expressed genes (DEGs) were mapped to Gene Ontology (GO) and Kyoto Encyclopedia of Genes and Genomes (KEGG) for functional enrichment analysis to understand the functional classification system and biological functions of genes. The DEGs were inputted into a protein–protein interaction (PPI) network analysis using STRING web server (http://www.string-db.org/).

### Validation of Gene Expression by Quantitative Real-Time Polymerase Chain Reaction

To validate the accuracy and reliability of RNA-seq data, the qRT-PCR was performed using the TB Green® Premix Ex Taq (TAKARA, Japan). The glyceraldehyde-3-phosphate dehydrogenase gene (*GAPDH*) gene was set as reference gene. The procedure was as follows: 95°C for 30 s, 95°C for 5 s, and 60°C for 20 s. Steps 2–3 were repeated for 40 cycles. The primers designed for qRT-PCR are shown in Supplementary Table 2. The relative expression was calculated by the 2^−ΔΔCt^ method.

### 16s rDNA Sequencing of Cecal Gut Microbiota

Fecal samples were collected and stored at −80°C until use (*n* = 6). Genomic DNA was extracted using the InviMag Stool DNA kit (Invitek, Berlin, Germany) and stored at −80°C for 16s ribosomal DNA (16s rDNA) gene sequencing. The V3–V4 region of the bacterial 16s rDNA was amplified by PCR using forward primer (5′-ACTCCTACGGGAGGCAGCA-3′) and reverse primer (5′-GGACTACHVGGGTWTCTAAT-3′) using Q5^®^ High-Fidelity DNA Polymerase (NEB, England). For each sample, the V3–V4 region of the bacterial was sequenced using the Illumina MiSeq platform (Illumina, USA). Shanghai Personal Biotech Co., Ltd., Shanghai, China, carried out the amplification and sequencing of the V3–V4 region.

### Statistical Analysis

The 16S rDNA sequencing data were analyzed using a linear discriminant analysis effect size to detect significant differences among the different groups. Spearman's correlation analysis among gut microbiota and other gene expression was evaluated using the genescloud tools (https://www.genescloud.cn). All data are presented as the mean ± standard deviation (SD) and compared by one-way ANOVA and *t*-test using the SPSS 25.0 software (Chicago, USA) between the experimental groups, and the graphs were drawn using the Prism 8.0 software. *p* < 0.05 was considered significant, and *p* < 0.01 was considered extremely significant.

## Results

### Effects of PFF on Body Weight and Carcass Weight

To determine the effect of PFF on the change of weight, the BW and weight gain were detected (Table 1). At the end of the experiment (90 days), the results indicated that the BW gain in the 25 and 35% PFF group was significantly lower than that in the control group (*p* < 0.05), whereas the carcass weight in the 25 and 35% PFF group was not significantly different from the control group (*p* > 0.05).

**Table 1:** Effect of probiotic fermented feed on body weight and carcass weight in ducks.

	**Control**	**25%PFF**	**35%PFF**
Body weight (kg)	1.31 ± 0.16^a^	1.19 ± 0.10^b^	1.19 ± 0.08^b^
Carcass weight (kg)	1.24 ± 0.16	1.22 ± 0.33	1.12 ± 0.08

*Data are expressed as mean ± SD (n = 9). Different superscript letters indicate significant differences between groups*

a, b *(p ≤ 0.05)*.

### Effects of PFF on Serum Lipid Parameters and Fat Accumulation

**Figure 1:**
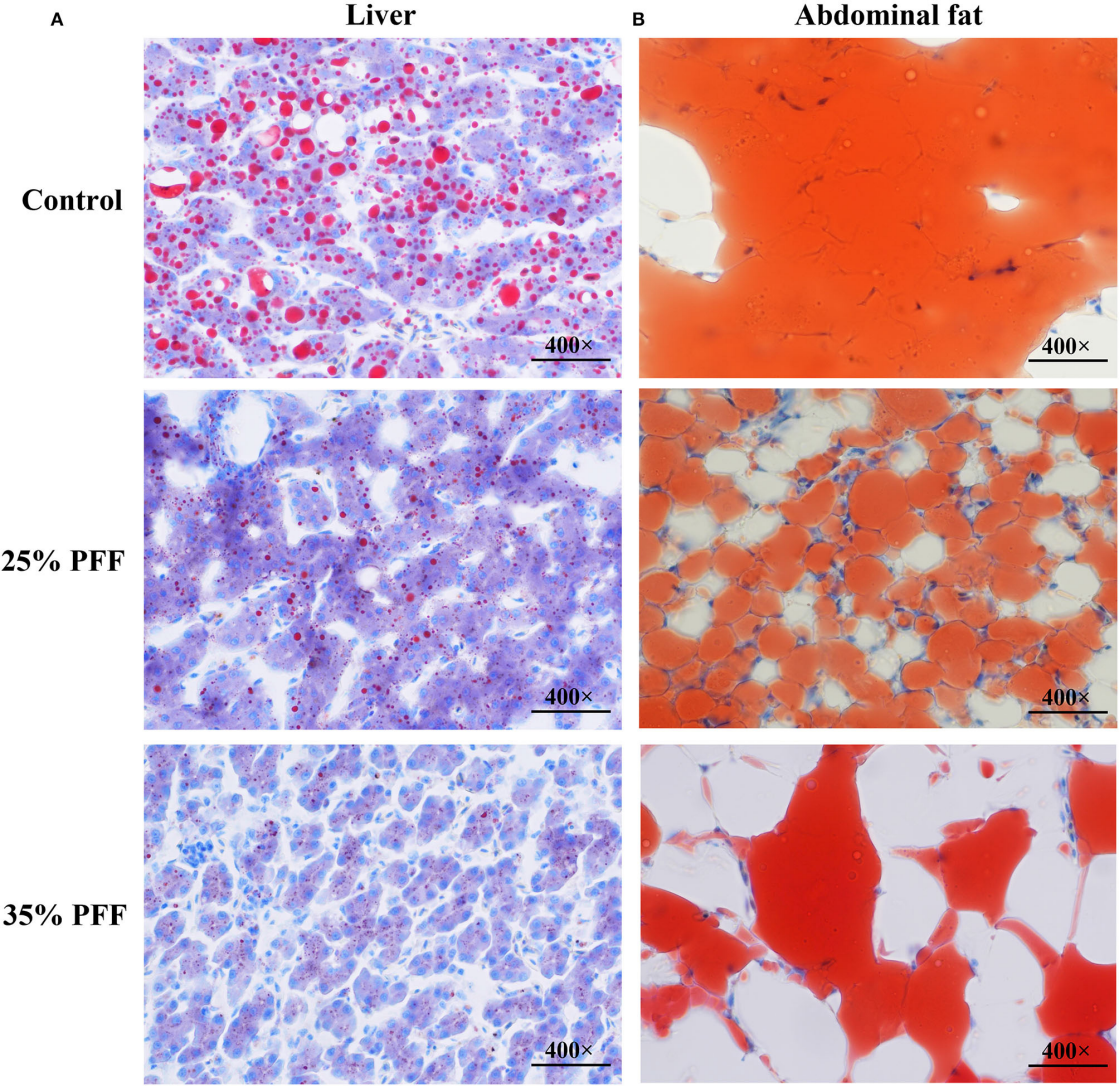
Effect of PFF on fat accumulation in Shaoxing ducks with Oil Red O staining. **(A)** Liver; **(B)** abdominal fat. The red color indicates lipid droplet, and the blue color indicates nucleus.

To explore the impact of PFF on lipid parameters in Shaoxing ducks, the levels of serum lipid indices (TG, TC, LDL-C, and HDL-C) were detected (Table 2). The results showed that there were no significant differences (*p* > 0.05) in TC, HDL-C, and LDL-C on day 210 among the three treatment groups, while the ducks fed 25 and 35% PFF showed lower TG compared to the control group (*p* < 0.05). To further evaluate the effect of PFF on duck fat deposition, the Oil Red O staining was tested in this study. As shown in Figure 1, lipid droplet area in 25 and 35% PFF group was smaller than that in the control group.

**Table 2:** Effect of probiotic fermented feed on serum lipid parameters in ducks.

	**Control**	**25%PFF**	**35%PFF**
TC (mmol/L)	6.61 ± 1.24	5.41 ± 0.56	6.98 ± 2.20
TG (mmol/L)	11.10 ± 5.33^a^	1.91 ± 0.90^b^	4.26 ± 1.57^b^
HDL-C (mmol/L)	2.53 ± 0.86	2.71 ± 0.16	2.75 ± 0.80
LDL-C (mmol/L)	3.14 ± 1.09	2.19 ± 0.88	1.91 ± 1.49

*TC, total cholesterol; TG, triglycerides; HDL-C, high-density lipoprotein cholesterol; LDL-C, low-density lipoprotein cholesterol*.

*Different superscript letters indicate significant differences between groups*

a, b *(P ≤ 0.05)*.

### Effect of PFF on Fecal Microbiota Composition

**Figure 2:**
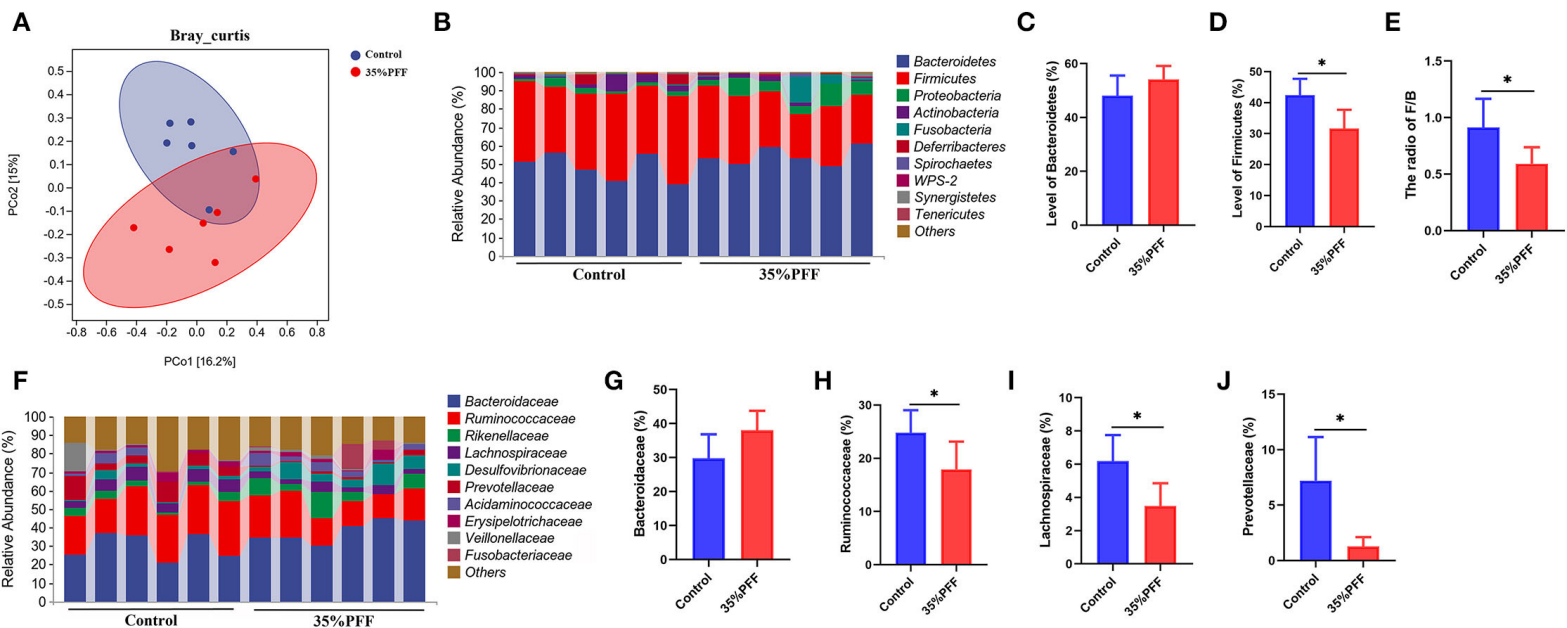
Effects of PFF on gut microbiota in Shaoxing ducks. **(A)** PcoA score plot. **(B)** Phylum-level distribution of fecal microbiota. **(C,D)** Relative abundance of the phyla Firmicutes and Bacteroidetes. **(E)** The ratio of Firmicutes/Bacteroidetes (F/B) levels. **(F)** Family-level distribution of fecal microbiota. **(G–J)** The relative abundance of Bacteroidaceae, Ruminococcaceae, Lachnospiraceae, and Prevotellaceae. *p < 0.05.

A total of 1,671,462 high-quality clean reads were generated from the 12 fecal samples. To evaluate differences in community microbiome structure between the control and 35% PFF groups, multiple diversity indexes were conducted. The results showed that the β-diversity exhibited significant separation of the principal components between the two groups (*p* = 0.005; Figure 2A), yet no significant difference was detected in the α-diversity (represented by Chao1, Faith_pd, Shanno, and Observed_species index) across groups (*p* > 0.05; Supplementary Figure 1), indicating that different interventions did not result in significant changes in taxa richness.

To further investigate the regulatory effects of PFF on the structure of the intestinal flora, the relative abundance of the predominant phylum and family was compared between the control and 35% PFF group (Figures 2B,F). At the phylum level, Firmicutes and Bacteroidetes were the leading phyla across all groups, occupying over 80% of the total sequences (*p* > 0.05; Figures 2C,D). The relative abundance of Firmicutes significantly decreased by feeding PFF supplementation (*p* < 0.05, Figure 2C), while the phylum Bacteroidetes showed no significance between the two groups (*p* > 0.05, Figure 2D). Interestingly, the Firmicutes/Bacteroidetes (F/B) ratio in the 35% PFF group was significantly lower than that in the control group (*p* < 0.05, Figure 2E). At the family level (Figure 2F), the relative abundance of Ruminococcaceae, Lachnospiraceae, and Prevotellaceae decreased significantly in the intestinal tract of 35% PFF ducks (*p* < 0.05, Figures 2G–I), but the family Bacteroidaceae showed no significance between the two groups (*p* > 0.05, Figure 2J).

### Transcriptome Analysis Revealed the Signal Transduction Pathway of PFF Affecting Shaoxing Ducks

**Figure 3:**
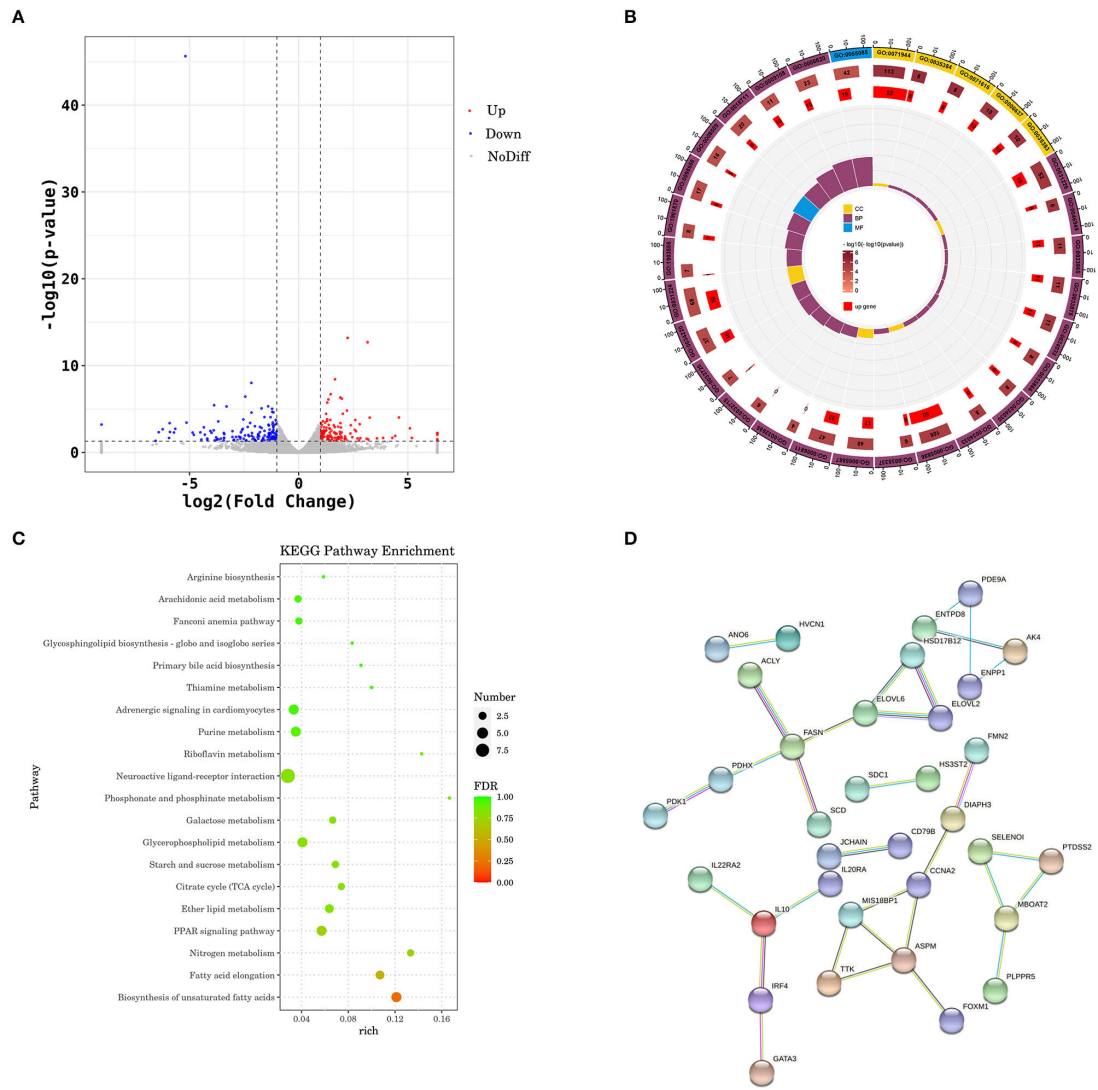
Transcriptomics analysis of liver on PFF treatment. **(A)** Volcano plot for expression comparisons between PFF and control ducks in liver samples. Red points indicate the significantly differentially upexpressed genes [false discovery rate (FDR) < 0.05], and blue points indicate the significantly differentially downexpressed genes (FDR < 0.05), while gray points indicate the genes with no significant differences. **(B)** GO enrichment circle diagram. **(C)** KEGG pathway. **(D)** The subnetwork of the PPI analysis.

A total of 131,569,140 and 133,028,546 were generated from the control and PFF, respectively. After adapter cleaning, quality trimming, and duplicate and length filtering, 89.98–91.54% of the clean reads were mapped to *A. platyrhynchos* genome (PK_2015) (Supplementary Table 3). A total of 291 DEGs (139 upregulated genes and 152 downregulated genes) were identified between the PFF group and the control group with FC > 2 and *p* < 0.05 (Figure 3A; Supplementary Figure 2). Significantly downregulated genes were involved in fatty acid synthase, e.g., fatty acid synthase (*FASN*), peroxisome proliferator-activated receptor gamma (*PPARG*), acyl-CoA desaturase (*SCD*), hydroxysteroid 17-beta dehydrogenase 12 (*HSD17B12*), ELOVL fatty acid elongase 2 (*ELOVL2*), ELOVL fatty acid elongase 6 (*ELOVL6*), and ATP citrate lyase (*ACLY*).

The DEGs in the PFF group were enriched for 720 GO terms (Supplementary Table 4). The top 30 GO terms of differences under each functional classification were enriched (Figure 3B), mainly involved in the response to fatty acid synthesis and metabolism, such as “acyl-CoA biosynthetic process” (GO:0071616), “acyl-CoA metabolic process” (GO:0006637), “fatty-acyl-CoA biosynthetic process” (GO:0046949), fatty-acyl-CoA metabolic process (GO:0035337), and fatty acid derivative biosynthetic process (GO:1901570).

The KEGG pathway enrichment analysis was subjected to predict the significantly enriched pathways (Figure 3C; Supplementary Table 5). Interestingly, the DEGs were mainly significantly enriched in lipid metabolism-related pathways, including fatty acid elongation (apla00062; *p* = 0.012; Supplementary Figure 3A), PPAR signaling pathway (apla03320, *p* = 0.033; Supplementary Figure 3B), and ether lipid metabolism (apla00565, *p* = 0.047; Supplementary Figure 3C).

The DEG PPI analysis was constructed using the STRING database to investigate the interactions between DEGs, and those that scored ≥0.07 were selected. Subsequently, the PPI of fatty acid synthesis and metabolism-related genes was obtained, including *FASN, SCD, ELOVL6, ELOVL2*, and *HSD17B12* (Figure 3D).

### qRT-PCR Validation of the DEGs

**Figure 4:**
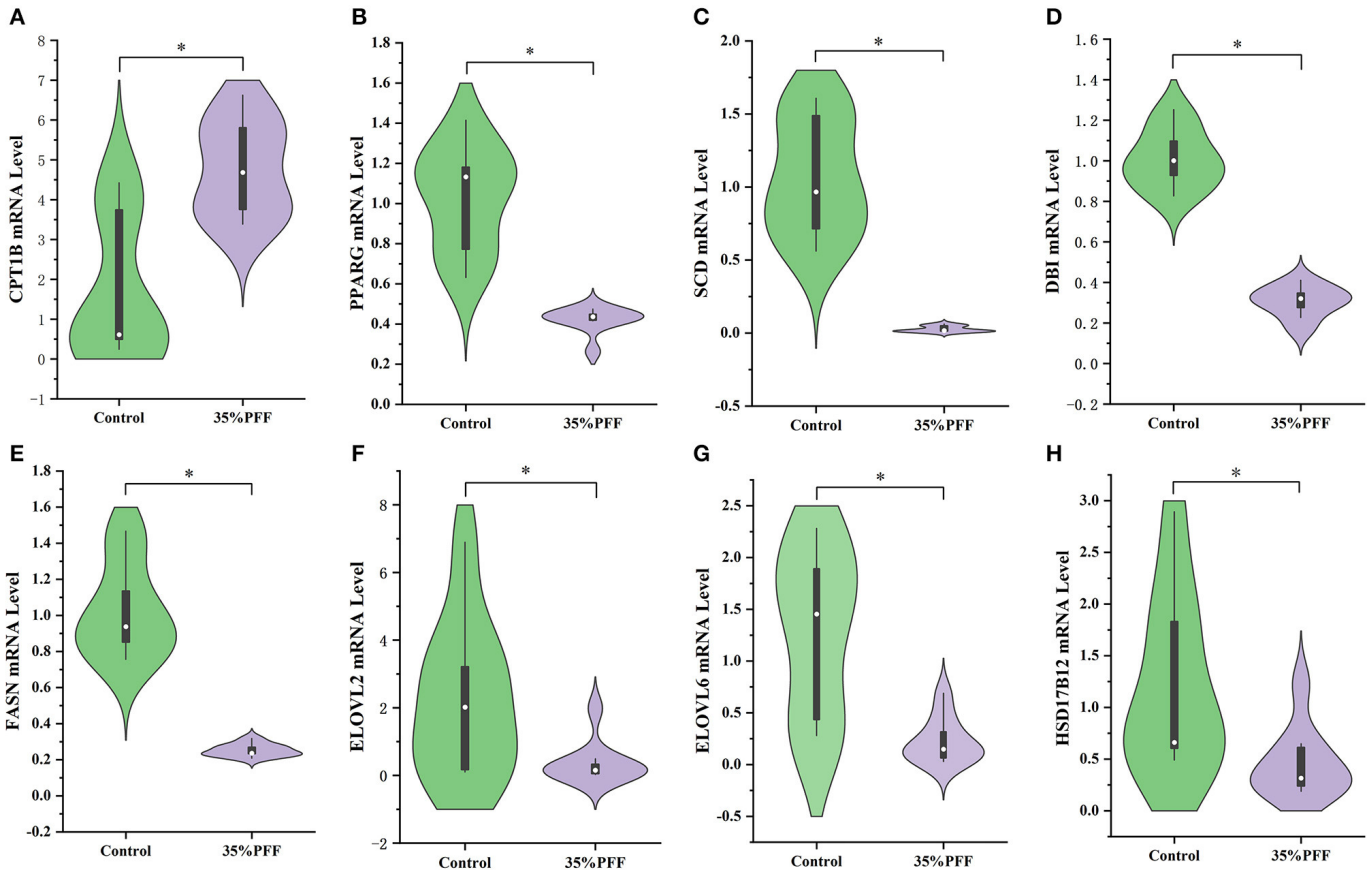
Results from quantitative real-time PCR (qRT-PCR) validation. **(A)** CPT1B. **(B)** PPARG. **(C)** SCD. **(D)** DBI. **(E)** FASN. **(F)** ELOVL2. **(G)** ELOVL6. **(H)** HSD17B12. Significant expression difference between control group (denoted in green color) and 35% PFF group (denoted in purple color) verified by qRT-PCR of eight genes. *p < 0.05.

To confirm the DEGs of liver between control and 35% PFF group, the eight DEGs' expression were performed using qRT-PCR. We found significant decreases in the expression of *CPT1B, PPARG, SCD, DBI, FASN, ELOVL2, ELOVL6*, and *HSD17B12* (Figure 4), and the expression changes of these eight genes were consistent with the results from liver transcriptomes. Therefore, the above results of qRT-PCR indicated that the RNA-seq data were reliable and could be represented as relative expression levels of DGEs in the livers of Shaoxing ducks.

### Spearman's Correlation Analysis

**Figure 5:**
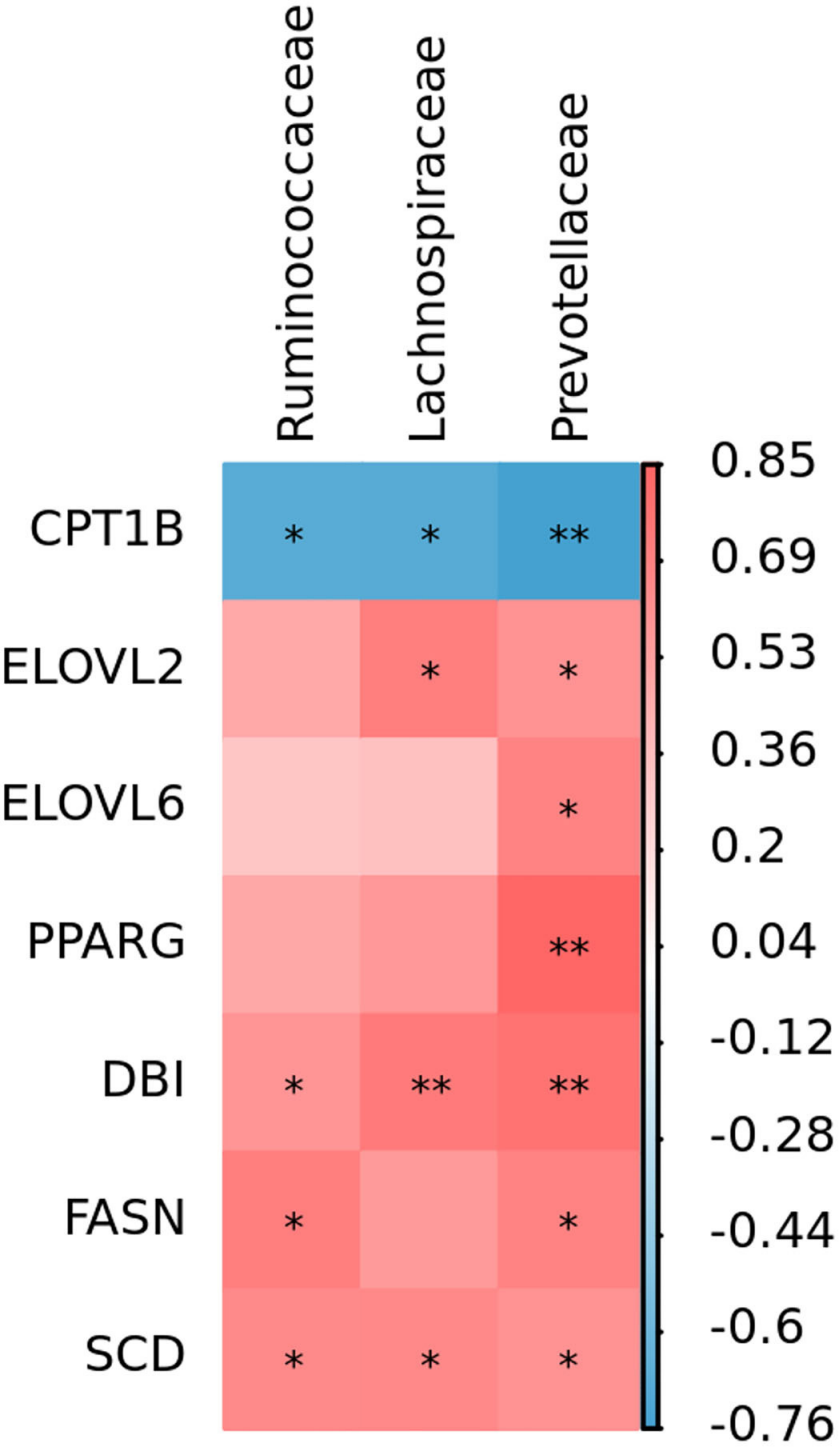
Spearman's analysis of PFF effect between gut microbiota and liver metabolism-related genes. *p < 0.05, **p < 0.01.

To investigate whether the liver fat degradation effects of PFF are related to changes in the gut microbiota, Spearman's correlation analysis was performed among the microbiota and liver metabolism-related genes. As presented in Figure 5, the hypoglycemic efficacy of PFF is also associated with the composition of the gut microbiota during the treatment of ducks.

## Discussion

The differentiation and lipid buildup of adipocytes are linked to the development of obesity, and excessive obesity may lead to the risk of fatty liver disease (Hermier, 1997). Some studies have demonstrated that fatty liver in birds could result in reducing production performance and increasing mortality (Fan et al., 2019). It is well-known that the degree of obesity can be directly expressed as the change in the liver index, and the blood lipid level can reflect the body's metabolic level. Increased level of TG in the blood is the main feature of NAFLD induced by HFD/F (Fan et al., 2019; Mu et al., 2020). In this study, supplementation of PFF exerted lipid-lowering effect by significantly decreasing the TG level and preventing the liver and abdominal fat accretion in ducks, while the TC, HDL-C, and LDL-C showed no significant change between the control and PFF groups, which indicated that PFF can significantly reduce fat formation.

Fermentation can improve the gut microbiota balance and mediate gut bacteria to prevent the development of obesity (Mu et al., 2020). Therefore, we elucidated the precise underlying mechanism of improved fat formation by PFF. Our results showed that PFF supplementation did significantly change the relative abundance of duck Firmicutes. Lower proportions of Firmicutes are helpful for the decomposition of fat deposition (Abdallah Ismail et al., 2011). Previous studies also showed that the F/B ratio is an important indicator for evaluating intestinal health and the lower the ratio, the greater the positive effect on the intestine (Bäckhed et al., 2004; Cheng et al., 2017). Our study revealed that PFF supplementation could significantly decrease the F/B ratio. Moreover, at the family level, after treatment with PFF, the richness of Ruminococcaceae, Lachnospiraceae, and Prevotellaceae decreased. Studies have also shown that the decrease in Ruminococcaceae, Lachnospiraceae, and Prevotellaceae could attenuate HFB-induced obesity effect (Serena et al., 2018; Zhang et al., 2020a), which is consistent with our findings.

An increasing amount of evidence indicates that using probiotics to affect the gut microbiome is a novel, safe, and effective specific liver treatment method to reverse the metabolic abnormalities of patients with obesity and ameliorate fat deposition (Kuno et al., 2016; Ma et al., 2019). Liver transcriptome and pathway analysis found that the expression of *PPARG, DBI, SCD, HSD17B12, ELOVL2, ELOVL6*, and *FASN*, involved in PPAR signaling pathway, fatty acid biosynthesis pathway, and fatty acid elongation pathway, was significantly decreased by PFF treatment; nevertheless, the expression of CPT1B was remarkably enhanced. PPARγ, as the key nuclear receptor, is considered to be an important target for regulating adipogenesis, and the downregulation of PPARγ expression would cause suppression of TG and stimulation of hepatic lipolysis level (Li et al., 2019). CPT1B, as the PPARγ signaling pathway downstream target gene, could accelerate the migration of fatty acids to mitochondria, and ultimately promote the oxidation of fatty acids in the liver (Ham et al., 2016). It has also been demonstrated that downregulation of CPT1B leads to impaired fatty acid oxidation (Vantaku et al., 2019). Furthermore, SCD and DBI (also named ACBP) are also main the members of PPARγ signaling pathway, which is responsible for fatty acid desaturation (Stoeckman and Towle, 2002; Neess et al., 2006). Meanwhile, besides the gene of PPARγ signaling pathway, other fatty acid-related genes, such as *FASN, HSD17B12, ELOVL2*, and *ELOVL6*, are also involved in lipid metabolism (Hachim et al., 2020; Maycotte-Cervantes et al., 2020; Wu et al., 2021). These data indicated that PFF might contribute to attenuate fatty acid synthesis, lipid generation, and accelerate the lipid metabolism to control hepatic lipid accumulation. Furthermore, Spearman's analysis revealed that the activation of the lipid metabolism-related gene expression may result from the decrease of gut bacteria by PFF. Thus, these findings revealed that the effect of PFF was associated with the modulation of the composition of gut microbiota and activation of the liver lipid metabolism-related genes.

## Conclusion

**Figure 6:**
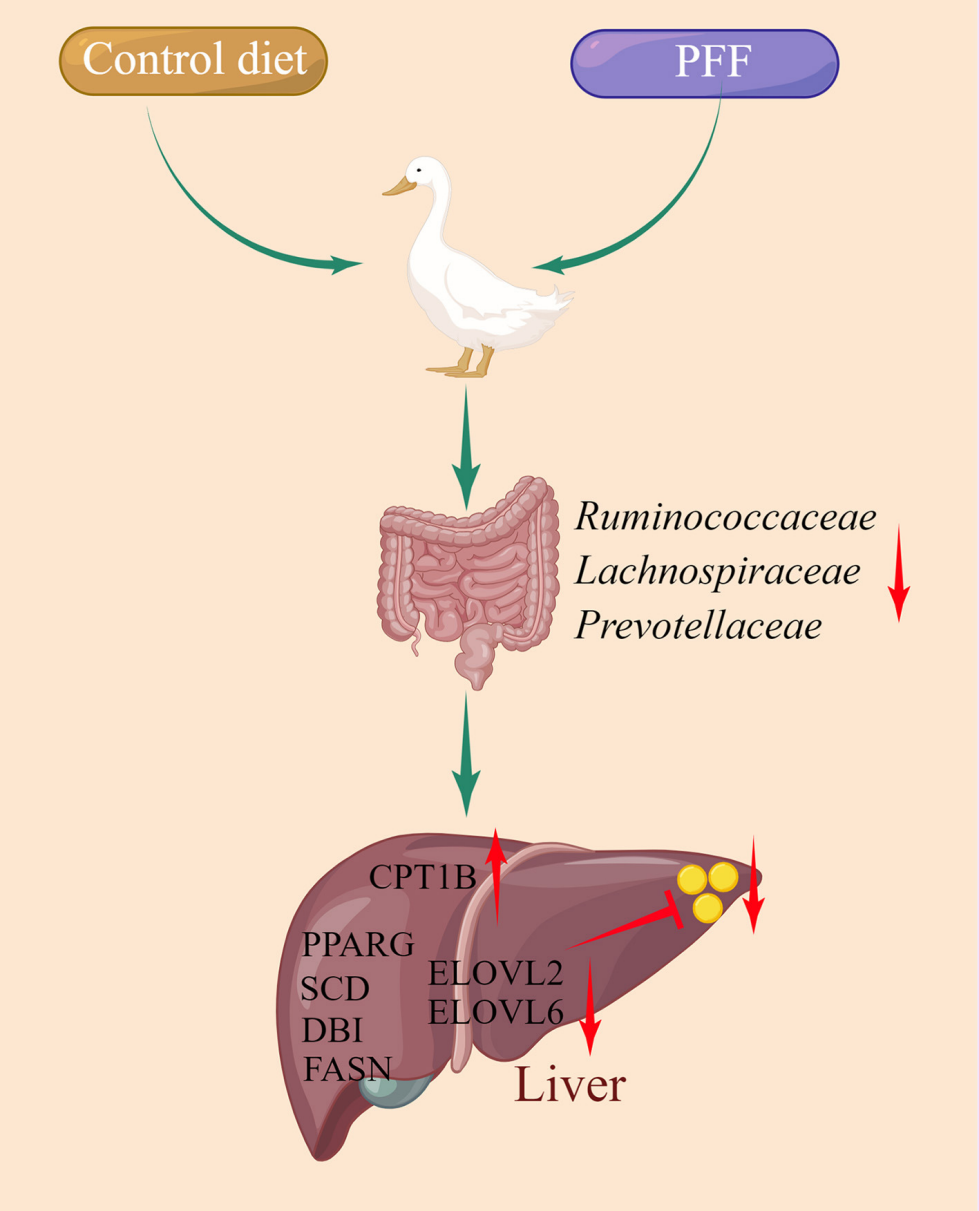
A model depicting use of PFF to alleviate liver fat deposition by targeting gut microbiota.

First, we demonstrated that PFF supplementation decreases serum TG level and reduces the formation of liver and abdominal lipid droplet. Second, PFF regulates the gut microbiota structure mainly by decreasing the F/B ratio and the relative abundance of bacteria, such as Ruminococcaceae, Lachnospiraceae, and Prevotellaceae. Third, PFF activates the production of lipid metabolism-related genes, such as *CPT1B, PPARG, DBI, SCD, HSD17B12, ELOVL2, ELOVL6*, and *FASN*. Overall, our findings indicate that PFF can alter the composition of gut microbiota to activate the lipid metabolism-related genes and reduce the formation of liver lipid droplet (Figure 6).

## Data Availability Statement

The datasets presented in this study can be found in online repositories. The names of the repository/repositories and accession number(s) can be found at: https://www.ncbi.nlm.nih.gov/, PRJNA825587 and https://ngdc.cncb.ac.cn/gsa/, CRA007254.

## Ethics Statement

All samplings were approved by the Zhejiang Academy of Agricultural Sciences' Animal Care Committee.

## Author Contributions

TG wrote this article. LL conceived of the study and participated in its design and coordination. RZ, WF, and CJ carried out animal experiment. TG and MD carried out serum lipid parameters determination, RNA purification, and quantitative RT-PCR. TG, TZ, WX, YT, and LC performed the transcriptomics and 16s rDNA analysis. TG and LL interpreted the results and contributed to editing the manuscript. All authors read and approved the final manuscript.

## Funding

This study was supported by the Zhejiang Science and Technology Major Program on Agricultural New Variety Breeding (2021C02068-10) and the China Agriculture Research System of MOF and MARA (CARS-42-6, CARS-42-39).

## Conflict of Interest

WF and CJ were employed by Jinhua Jinwu Agricultural Development Co., Ltd. The remaining authors declare that the research was conducted in the absence of any commercial or financial relationships that could be construed as a potential conflict of interest.

## Publisher's Note

All claims expressed in this article are solely those of the authors and do not necessarily represent those of their affiliated organizations, or those of the publisher, the editors and the reviewers. Any product that may be evaluated in this article, or claim that may be made by its manufacturer, is not guaranteed or endorsed by the publisher.
