# Supplementary material for: Probiotic Fermented Feed Alleviates Liver Fat Deposition in Shaoxing Ducks via Modulating Gut Microbiota
Source: Front Microbiol. 2022 Jul 13;13:928670. doi: 10.3389/fmicb.2022.928670 (PMC9326468; doi:10.3389/fmicb.2022.928670)
Supplement: Supplementary file 1 [file Table_1.DOCX]

Table S1. The composition and nutrient levels of the experimentaldiets.

| Items | | | |
| --- | --- | --- | --- |
| Ingredient (%) |  | Nutrion levels^2^ |  |
| Corn | 53.2 | Metabolic energy（MJ/kg） | 1.00 |
| Soybean meal | 25.8 | Crude protein | 17.00 |
| Wheat bran | 10.414 | Calcium | 3.6 |
| CaHPO_4_ | 1.35 | Total phosphorus | 0.74 |
| Limestone | 7.8 | Available phosphorus | 0.35 |
| DL-methionine | 0.136 | Lysine | 0.87 |
| Nacl | 0.30 | Methionine | 0.40 |
| Premix^1^ | 1.00 | Methionine＋Cystine | 0.72 |
| Total | 100 |  |  |

^1^Premix supplied the following per kilogram of diet: VA 12000 IU,VD_3_ 1800IU, VE 26 mg, VK_3_ 1.00 mg, VB_1_ 3.0 mg, VB2 9.60 mg, VB_6_ 6.00 mg, VB_12_ 0.03 mg, calcium pantothenate 28.5 mg, folic acid 0.60 mg, biotin 0.15 mg, Fe 50 mg, Cu 10 mg, Mn 90 mg, Zn 90 mg, I 0.50 mg, Se 0.40 mg.

^2^Crude protein, calcium and total phosphorus were measured values, and the rest were calculated values.
