# Supplementary material for: Probiotic Fermented Feed Alleviates Liver Fat Deposition in Shaoxing Ducks via Modulating Gut Microbiota
Source: Front Microbiol. 2022 Jul 13;13:928670. doi: 10.3389/fmicb.2022.928670 (PMC9326468; doi:10.3389/fmicb.2022.928670)
Supplement: Supplementary file 2 [file Table_2.DOCX]

**Supplementary Table 2. The primer information of qRT-PCR**

| **Gene** | **Primer (5' to 3')** |
| --- | --- |
| CPT1B | GCTGATGGACGACGAGAAGT |
|  | ACCAGTCGCTCACGTAGTTG |
| HSD17B12 | AGCCAAGCTTCGATAAGCCC |
|  | TGAGAAGACCCAGCCCATGA |
| ELOVL2 | TCATGAAGAACAGGCCTCCG |
|  | ACCTTGGCTACCCGGATGT |
| SCD | ACCATAGGGCTCAATGCCAC |
|  | TGGAAGCCTTCTCCTAGGGC |
| ELOVL6 | CTCAGAGGAGGCATTTCA |
|  | GCTCGCTTGTTCATTAGG |
| DBI | AGGTGAAGCAGCTCAAGTCG |
|  | ATACCAGGGCGATCCGTGT |
| PPARG | ACTTGACAGCACCAGGCATT |
|  | TCCATCGCAGACAGATCCAC |
| FASN | AGGGAGACAGTGAGGAGAGC |
|  | ACTTCTCCCTGTTGCTGCAC |
| GAPDH | GGTTGTCTCCTGCGACTTCA |
|  | TCCTTGGATGCCATGTGGAC |
