# Supplementary material for: Probiotic Fermented Feed Alleviates Liver Fat Deposition in Shaoxing Ducks via Modulating Gut Microbiota
Source: Front Microbiol. 2022 Jul 13;13:928670. doi: 10.3389/fmicb.2022.928670 (PMC9326468; doi:10.3389/fmicb.2022.928670)
Supplement: Supplementary file 3 [file Table_3.DOCX]

**Supplementary Table 3. Summary statistics for sequence quality and alignment information of different liver samples.**

| **Group** | **Sample** | **Raw reads** | **Clean reads** | **Q20** | **Q30** | **Total mapped** | **Uniquely mapped** | **Multiple mapped** |
| --- | --- | --- | --- | --- | --- | --- | --- | --- |
| **Control** | CK1 | 39,562,792 | 36,362,886 | 97.20% | 92.93% | 33,052,604 (90.90%) | 3,234,819  (9.79%) | 29,817,785 (90.21%) |
|  | CK2 | 43,536,284 | 40,053,912 | 97.31% | 93.20% | 36,039,409 (89.98%) | 3,457,123  (9.59%) | 32,582,286 (90.41%) |
|  | CK3 | 48,470,064 | 44,052,148 | 97.36% | 93.30% | 39,973,580 (90.74%) | 3,579,108  (8.95%) | 36,394,472 (91.05%) |
| **35%** **probiotic fermented feed (PFF)** | PFF1 | 51,014,660 | 46,110,784 | 97.32% | 93.28% | 41,535,048 (90.08%) | 5,251,714  (12.64%) | 36,283,334 (87.36%) |
|  | PFF2 | 42,459,616 | 38,774,212 | 97.18% | 92.95% | 35,056,371 (90.41%) | 3,921,812  (11.19%) | 31,134,559 (88.81%) |
|  | PFF2 | 39,554,270 | 36,355,228 | 97.26% | 93.11% | 33,280,158 (91.54%) | 3,503,347  (10.53%) | 29,776,811 (89.47%) |
