# Supplementary material for: Probiotic Fermented Feed Alleviates Liver Fat Deposition in Shaoxing Ducks via Modulating Gut Microbiota
Source: Front Microbiol. 2022 Jul 13;13:928670. doi: 10.3389/fmicb.2022.928670 (PMC9326468; doi:10.3389/fmicb.2022.928670)
Supplement: Supplementary file 6 [file Data_Sheet_1.docx]

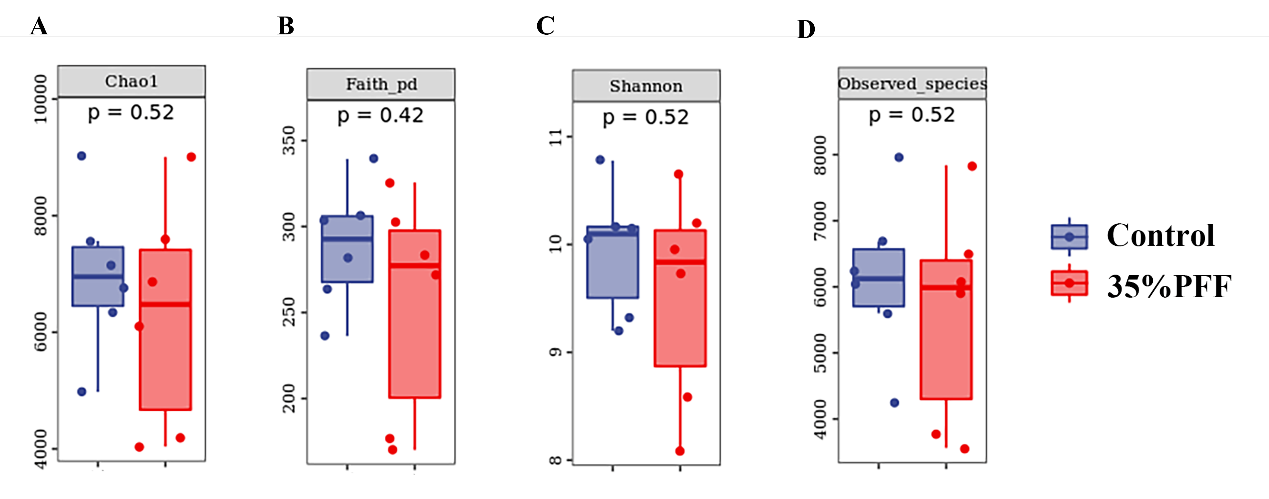


**Supplementary Figure 1** **Alpha diversity.** (A) Variations in alpha diversity between 35%PFF and control. Comparison of 16S in chao1 index (A), faith_pd index (B), Shannon index (C) and Observed_species index (D).


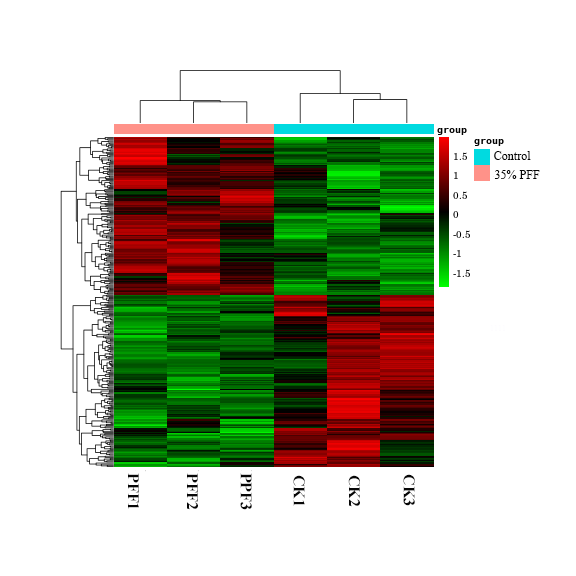


**Supplementary Figure 2 Heat map plot of DEGs in liver tissues between control group (CK1, CK2 and CK3) and 35% PFF (PFF1, PFF2 and PFF3) using TPM expression value of genes by adopting hierarchical clustering method.** Each column represents a specimen and each row represents a gene. Red color indicates genes that were up-regulated and blue color indicates genes that were down-regulated.


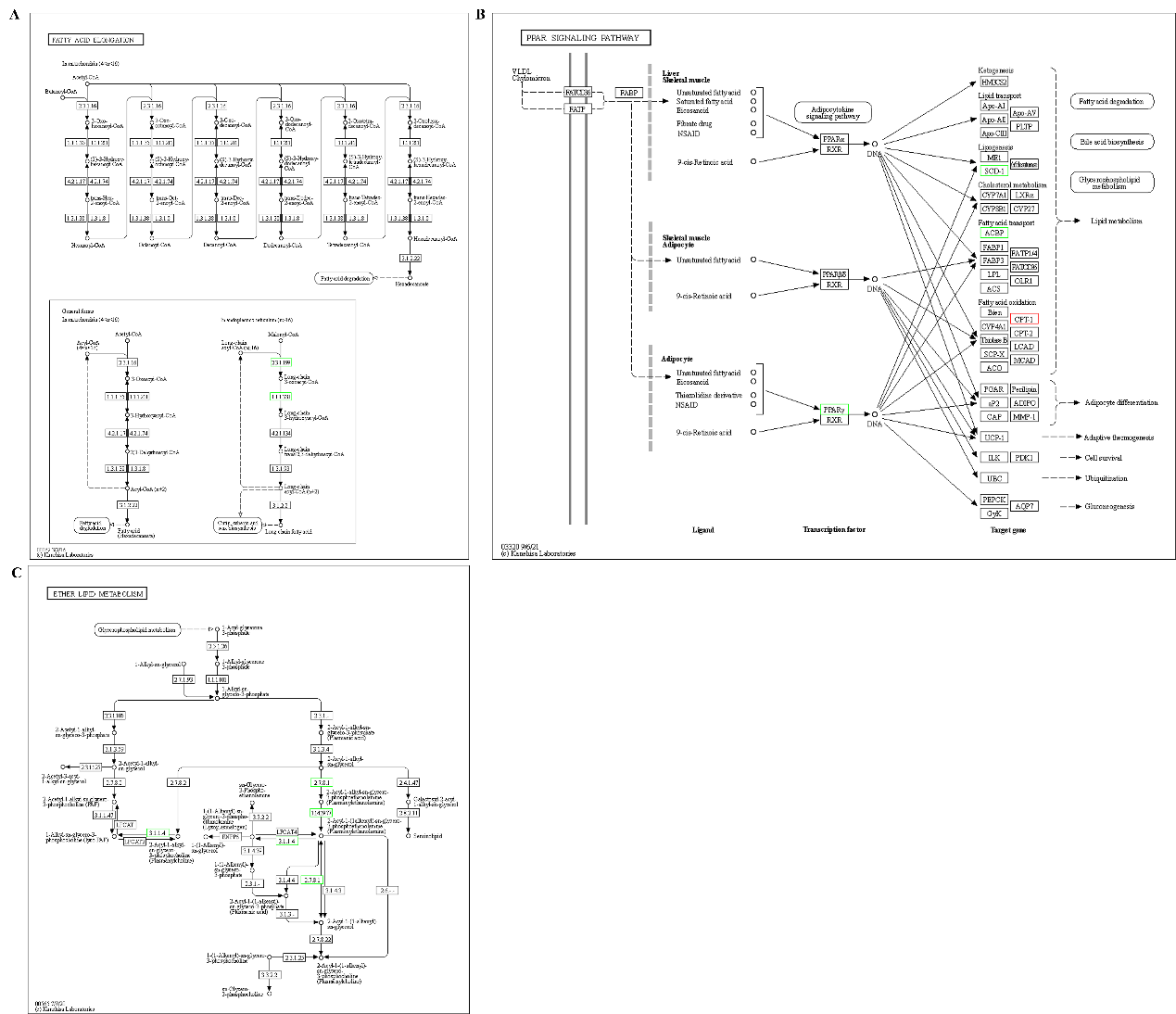


**Supplementary Figure 3 Differentially expressed genes between 35% PFF and control group in pathways.** (A) Fatty acid elongation pathway. (B) PPAR signialing pathway. (C) ether lipid metabolism. Significantly differentially up-regulated genes are in red font and significantly differentially down-regulated genes are in green font.
